# 

**Published:** 1881-10

**Authors:** 


SURG GENLS OFFS

Vol. XVIII. October 1881. No. 3.

NOTHING TO INDEX

### The Bistoury.

Thad S. Up de Graff. M.D.

